# Microscopic Identification, Phytochemical Analysis, and Study of Antioxidant Properties of Branches, Leaves, and Fruits of Kazakh Medicine *Sambucus sibirica*

**DOI:** 10.3390/molecules29235503

**Published:** 2024-11-21

**Authors:** Pengyan Yan, Shuak Halimubek, Jingjing Chen, Wenhuan Ding, Sien Fan, Dongdong Wang, Xiaoqing Zhang, Haiyan Xu, Xuejia Zhang

**Affiliations:** 1 College of Traditional Chinese Medicine, Xinjiang Medical University, Urumqi 830017, China; 17590823073@163.com (P.Y.); xuah0529@163.com (S.H.); 17799160495@163.com (J.C.); baggio19891988@163.com (S.F.); wang_tcm@126.com (D.W.); 15699091569@163.com (X.Z.); xhy66003@163.com (H.X.); 2 Central Laboratory, Xinjiang Medical University, Urumqi 830054, China; dingding9089@sina.com; 3 Xinjiang Laboratory of Famous Prescription and Science of Formulas, Xinjiang Medical University, Urumqi 830017, China; 4 Xinjiang Key Laboratory of Planting Standards for Authentic and Superior Chinese Medicinal Materials, Xinjiang Medical University, Urumqi 830017, China

**Keywords:** *Sambucus sibirica*, microscopic identification, FTIR, TLC, UPLC-QqQ-MS/MS, antioxidant

## Abstract

*Sambucus sibirica*, a deciduous shrub from the Adoxaceae family, is a traditional Kazakh medicine used in Xinjiang, China. Its branches, leaves, and fruits are used to treat fractures, rheumatoid arthritis, and nephritis. To advance research on *S. sibirica*, we conducted studies on its microscopic identification, chemical composition, and biological activity. The cross-sectional features of the branches, leaves, and fruits were observed under a microscope, revealing different types of ducts, cork cells, non-glandular hairs, oil droplets, stone cells, scale hairs, and star-shaped hairs in the *S. sibirica* powders. Fourier transform infrared spectroscopy (FTIR) was used to characterize the presence of specific chemical groups, revealing similarities and differences between different parts. Thin-layer chromatography (TLC) confirmed that chlorogenic acid was present in the branches, leaves, and fruits, whereas rutin was more prominent in the leaves. The total flavonoid contents were determined by a photocolorimetric approach and resulted in values of 7419.80, 5193.10, and 3629.10 μg·g^−1^ (dry weight) for the leaves, branches, and fruits, respectively. Further qualitative and quantitative analyses via ultra-performance liquid chromatography coupled with triple quadrupole tandem mass spectrometry (UPLC-QqQ-MS/MS) identified rutin, chlorogenic acid, quercetin, isoquercetin, and astragalin, with contents ranging from 1.00 to 4535.60 μg·g^−1^ (dry weight). Antioxidant tests revealed that the branches, leaves, and fruits of *S. sibirica* presented antioxidant properties, with the leaves demonstrating the highest activity, followed by the branches and fruits. These results align with the results of the quantitative analysis. This study provides valuable insights into the microscopic features, chemical composition, and antioxidant activity of *S. sibirica*, laying the foundation for its pharmacognosy research and quality standards and offering a reference for its future development and utilization.

## 1. Introduction

The genus *Sambucus* L. belongs to the family Adoxaceae and comprises approximately 30 recognized species worldwide. Certain species, such as *S. nigra*, *S. javanica*, *S. canadensis*, *S. williamsii*, and *S. adnate*, have been utilized in traditional medicine to treat various conditions, including bone fractures, rheumatism, influenza, diabetes, respiratory and pulmonary disorders, skin diseases, inflammatory ailments, and diarrhea [1]. Typically, their branches, leaves, flowers, and fruits serve as medicinal parts [2]. Additionally, the flowers and fruits are edible and have been prepared as popular beverages in Europe to prevent influenza and other ailments [1]. In traditional Chinese medicine, *Sambucus* plants are utilized primarily for bone-related issues and protection [3]. They contain a diverse range of bioactive phytochemical components, including phenolic compounds, terpenoids, fatty acids, cyanogenic glycosides, phytosterols, lectins, organic acids, alkaloid, coumarin, anthraquinone, and others [1]. These components exhibit a variety of significant pharmacological activities, including antibacterial, antifungal, antiviral, antidiabetic, anti-inflammatory, analgesic, antidepressant, anti-giardial, bone-protective, antioxidant, immunomodulatory, antiulcerogenic, and wound-healing properties [1].

In China, five *Sambucus* species have been identified and are commonly used for medicinal purposes. Among these, *S. sibirica* is recognized as a traditional Kazakh medicine found in the Altai Mountains of Xinjiang, as well as in neighboring regions of Russia, Mongolia, and Kazakhstan. Some scholars believe that *Sambucus racemosa* subsp. *sibirica* (Nakai) H. Hara [4], or *S. racemosa* subsp. *sibirica*, is homotypic synonym of *S. sibirica* [5]. The branches and leaves of *S. sibirica* are primarily used to treat fractures, falls, injuries, rheumatoid arthritis, and nephritis [6]. Recent studies have indicated that *S. sibirica* might enhance the bone fracture healing process and contains specific flavonoids and triterpenes [7,8]. However, limited modern research has been conducted on *S. sibirica*, and it remains uncertain whether its fruit possesses medicinal or edible applications like those of other *Sambucus* species. Consequently, systematic research on the available parts of *S. sibirica* is still lacking. Further pharmacognostic, phytochemical, and bioactive investigations are necessary to establish its identification and quality standards, ensuring its safe and effective use.

In general, different medicinal plant parts possess varying chemical compositions, which can result in differences in efficacy [9]. By developing medicinal plant resources that leverage the unique chemical compositions and pharmacological activities of different plant parts, we can increase the efficiency of sourcing medicinal materials. For example, the roots of certain *Rubia* species contain quinones, cyclopeptides, and triterpenoids, which are utilized in the treatment of various conditions, such as bleeding, cardiovascular diseases, tuberculosis, rheumatism, arthralgia, urinary calculi, inflammation, and skin infections [10]. Additionally, they serve as natural red-orange dyes. The aerial parts of the *Rubia* plant contain anthraquinones, phenolic acids, and tannins, which have been developed into granules for treating childhood diarrhea [11]. The fruits of these plants are rich in anthocyanins and polyphenolic compounds, suggesting their potential applications as antioxidants and food coloring agents [12]. Even the metabolites produced by endophytes hold potential as new drug resources [13].

Therefore, various medicinal plant parts of *S. sibirica* should be subjected to pharmacognostic, phytochemical, and bioactive investigations. Microscopy plays a crucial role in establishing quality standards for medicinal materials by highlighting differences in microscopic features, which can also assist in distinguishing related varieties [14]. Multiple pharmacopeias and medicinal standards emphasize the crucial role of microscopic identification in the establishment of quality standards and in ensuring the authenticity and quality of medicinal materials. Transverse section and powder identification can observe microstructural features, which are critical for accurate identification and differentiation. Chemical authentication can be conducted using spectroscopic and chromatographic methods, taking advantage of differences in chemical composition [15]. A quantitative analysis can provide insights into the distribution and concentrations of key active ingredients in medicinal materials, facilitating quality standardization [16]. Additionally, bioactivity experiments validated the effectiveness and therapeutic potential of these materials [17]. These findings could support the quality standardization of *S. sibirica* and promote the utilization of its various medicinal parts. The present research aimed to examine and compare the microscopic, chemical, and bioactive properties of the branches, leaves, and fruits of *S. sibirica.* The findings of this study will contribute to the field of pharmacognosy and assist in establishing quality standards for herbal products while also serving as a reference for their future development and utilization.

## 2. Results

### 2.1. Microscopic Characterization of S. sibirica

#### 2.1.1. Plant Morphology

**Figure 1:**
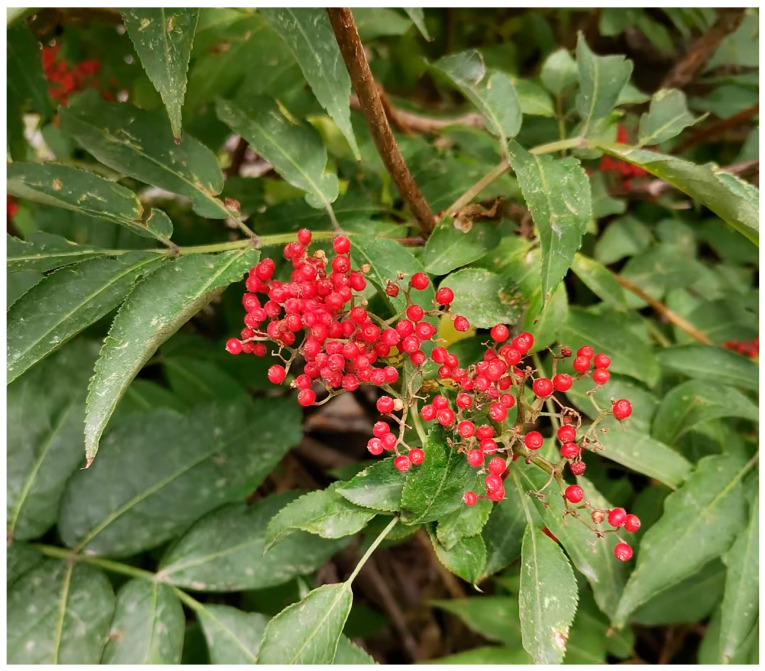
Pictures of *S. sibirica* plant.

As shown in Figure 1, *S. sibirica* is a deciduous shrub that reaches a height of 2 to 4 m. It features densely branched, light reddish-brown bark that is hairless. The leaves are feathered and consist of small ovate lanceolate or lanceolate leaflets with pointed tips and irregular sharp serrations along the edges, typically measuring 4.5 to 8.5 cm in length and 2 to 4 cm in width. These leaves are accompanied by glandular bracts. The conical inflorescences are upright, with corollas that are light green to light yellow, and rectangular lobes. The stamens are reddish yellow, whereas the fruit is bright red and spherical, measuring 4 to 6 mm in diameter. Each fruit contains two to three nuclei that are oval or elliptical with wrinkles and range from 2.5 to 3.5 mm in length. The flowering period occurs from April to May, and the fruiting period spans from June to August [18].

#### 2.1.2. Transverse Section

**Figure 2:**
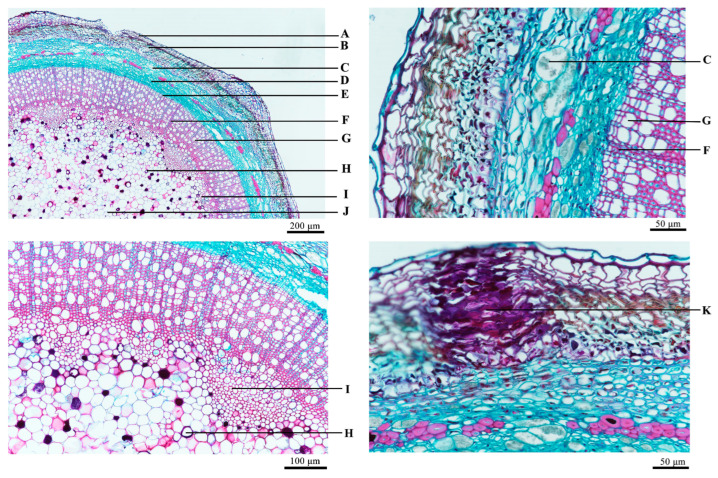
Microscopic view of branches. A: cork layer, B: cortex, C: clusters of calcium oxalate, D: phloem, E: formation layer, F: wood rays, G: xylem, H: tannin cells, I: polyprototypic primary xylem, J: pith, K: wood fibers.

Branch part: As shown in Figure 2, the epidermis comprises a single layer of brick-shaped cells that are neatly and closely arranged. The periderm consists of a corky layer, a cork-forming layer, and an inner layer of embolus, featuring square-like or irregularly shaped cells that are tangentially elongated. The cortex is primarily composed of thin-walled cells with clusters of calcium oxalate, and fiber bundles are intermittently arranged in a ring toward the inner side. The cells of the bast are arranged in an elongated strip and contain crystals. The rings of the formative layer are evident. The xylem is made up of conduits, wood fibers, and wood-thin-walled cells, including multiprototypical primordial xylem of various sizes arranged radially. The medulla is broad, occupying approximately half of the transverse section and containing a small proportion of conduits, with tannin cells dispersed throughout.

**Figure 3:**
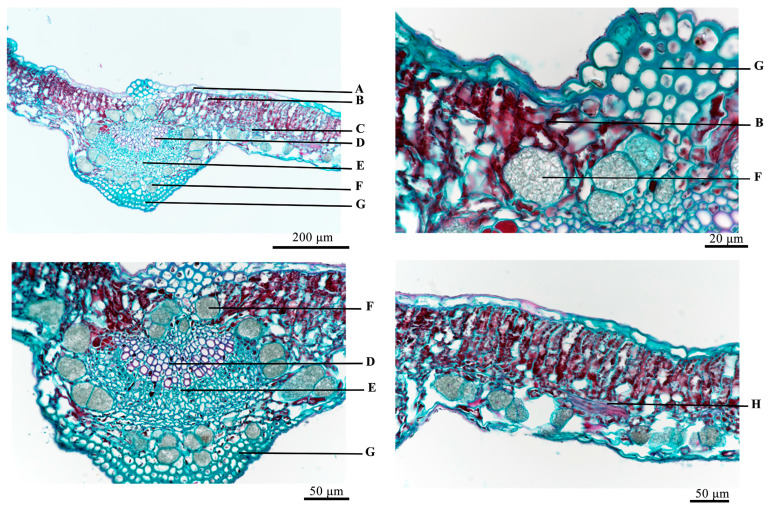
Microscopic view of leaves. A: upper epidermis, B: palisade tissue, C: sponge tissue, D: xylem, E: phloem, F: crystal sand, G: thick angle tissue, H: catheter.

Leaves: As shown in Figure 3, both the upper and lower epidermis consist of a single layer of tightly arranged cells, predominantly oblong, elliptic, or orbicular in shape, varying in size. The fenestrated tissue, located close to the upper epidermis, comprises three to four columns of cylindrical cells that are neatly arranged and contain numerous chloroplasts. The spongy tissue, situated near the lower epidermis, features irregularly shaped cells that are more sparsely arranged and contain fewer chloroplasts. The vascular bundles of the main vein, composed of xylem and phloem, are arranged in a fan shape, with the main veins having thick angular tissue composed of 8 to 10 columns of cells on the inner side. Thin-walled cells with clusters of crystal sand are common, and the innermost layer is arranged in a ring-like pattern that is intermittently present in the vascular bundles on the outside and spongy tissue, generally ranging from 10 to 60 μm.

**Figure 4:**
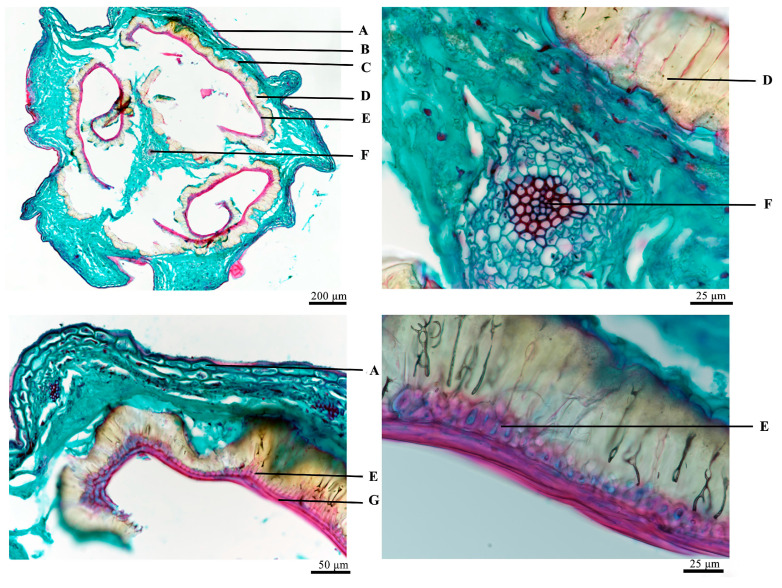
Microscopic view of fruits A: epicarp, B: mesocarp, C: endocarp, D: palisade cell, E: stone cell layer, F: vascular bundle, G: nutrient layer.

Fruit part: As shown in Figure 4, the epicarp is composed of 1–2 layers of closely arranged oblong or ellipsoid cells. The mesocarp is made up of thin-walled cells containing fine vascular bundles dispersed throughout. The endocarp, which represents the innermost layer of the pericarp, is either oblong or irregularly shaped. The palisade cell of the seed coat consists of a single column of narrowly arranged cells, characterized by significantly thickened lignified walls. The stony cell layer is distinctive, exhibiting either an oblong or square shape with tightly arranged cells. In contrast, the nutrient layer is a flattened, degraded thin layer.

#### 2.1.3. Powder Observation

**Figure 5:**
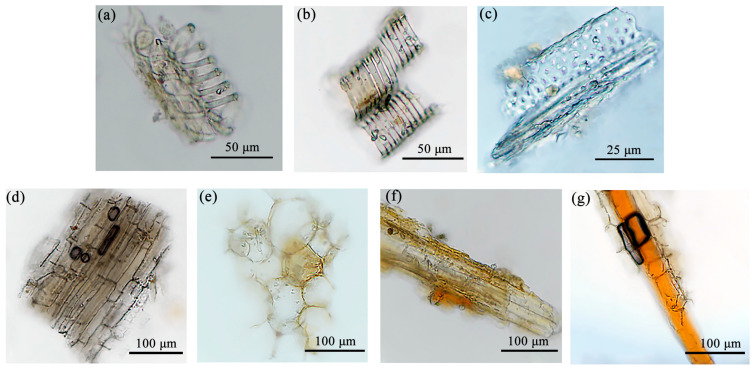
Powder characteristics of branches. (**a**) Spiral vessel, (**b**) staircase ducts, (**c**) marginal pore ducts, (**d**) wood ray cells, (**e**) wood cork cells, (**f**) wood fibers and crystals, (**g**) medullary secretion tracts.

Branches: The powder appears grayish brown. As illustrated in Figure 5, there is an abundance of spiral vessels and staircase ducts, whereas fewer ladder conduits are visible, predominantly with a diameter in the range of 20–40 μm. The staircase ducts exhibit significant fragmentation, with diameters ranging primarily from 20 to 50 μm. The wood ray cells are either square or irregularly shaped and feature tiny calcium oxalate single crystals on the fibers, which are elongated or spindle shaped. The surfaces of the cork cells are polygonal and vary in size. The medullary secretion tracts are rare and tubular in form, exhibiting reddish-brown pulp with diameters in the range of 20–45 μm.

**Figure 6:**
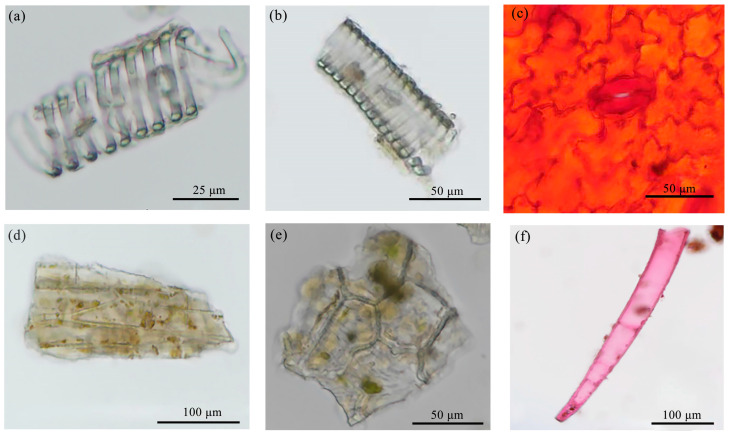
Powder characteristics of leaves: (**a**) spiral ducts, (**b**) trapezoidal ducts, (**c**) indeterminate stomata, (**d**) wood fibers, (**e**) epidermal cells, (**f**) non-glandular trichomes.

Leaves: The powder is dark green and contains common spiral and trapezoidal ducts with diameters predominantly between 25 and 55 μm (Figure 6). The epidermal cells are irregularly shaped, and the stomata are unevenly distributed. Non-glandular trichomes are sparse. The wood fibers are rectangular or spindle shaped, with diameters ranging primarily from 60 to 120 μm. Crystal clusters were observed in transverse sections, but no traces were found in the temporary slide of powder. When the leaves were grinded into powder, the periodic and ordered arrangement of the crystal clusters were disrupted. Aggregated small crystals are easily observable, but not in discrete situations. As a result, clustered single crystals are visible in the transverse section of *S. sibirica* leaves, while no scattered crystals were observed in the powder sample.

**Figure 7:**
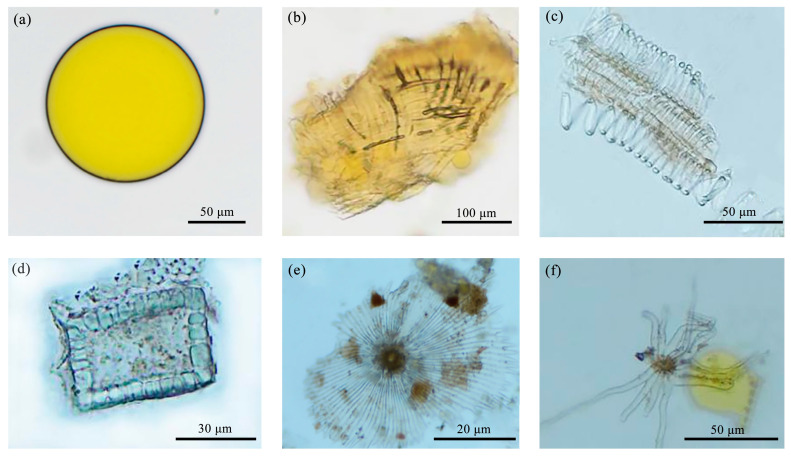
Powder characteristics of fruits: (**a**) oil droplets, (**b**) palisade mesophyll cells, (**c**) spiral ducts, (**d**) stone cells, (**e**) scaly trichomes, (**f**) stellate trichomes.

Fruit part: The powder is an orange-red color and is characterized by numerous oil droplets of varying sizes, with larger droplets reaching approximately 150 μm. The epidermal cells are irregular, and there are few spiral ducts and non-glandular trichomes (Figure 7). Stone cells occasionally present straight stems measuring 70–120 μm, appearing either square or round, with scaly trichomes ranging from 50 to 70 μm in diameter, along with a few stellate trichomes.

### 2.2. Fourier Transform Infrared (FTIR) Spectroscopy

**Figure 8:**
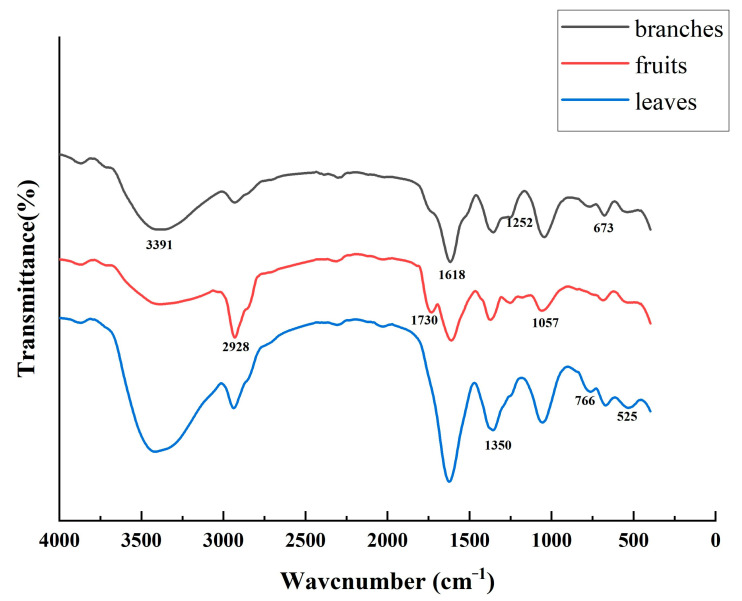
Infrared spectroscopy mapping of *S. sibirica* branches, leaves, and fruits.

The acquired data were processed using OriginPro 2021 software for basic plotting, baseline correction, and smoothing, allowing for the inference of functional groups and specific compound structures based on the position, intensity, and shape of the absorption peaks. The infrared absorption spectrum is presented in Figure 8. An analysis of the spectra revealed multiple strong absorption peaks in the range of 0–4000 cm^−1^ across the branches, leaves, and fruits of *S. sibirica*, indicating a complex chemical composition. Notably, the characteristic peaks of the three parts generally display similar patterns, with the peaks of the leaves being higher than those of the branches and fruits, suggesting a relatively higher contents of chemical components in the leaves.

In Figure 8, the peak at 3391 cm^−1^ is attributed to the stretching vibrations of the O-H bond. The double peaks at approximately 2928 cm^−1^ and 2828 cm^−1^ correspond to the stretching vibrations of methylene C-H. The absorption peak at 1730 cm^−1^ represents the C=O stretching vibrations of carbonyl groups, whereas the peak at 1618 cm^−1^ indicates the presence of C=C bonds. The peak at 1350 cm^−1^ is associated with C-H bending vibrations, and the peak at 1252 cm^−1^ corresponds to C-H deformation vibrations. The absorption peak at 1057 cm^−1^ is attributed to C-O stretching vibrations. Additionally, the peaks at 766, 673, and 525 cm^−1^ represent out-of-plane bending vibrations of the C-H bond. Notably, the absorption peak at 1730 cm^−1^ in the fruit sample is greater than that in the other parts, likely due to the stretching vibrations of C=O from lipids, indicating that this peak may serve as a characteristic peak for fruits, especially considering that the fruits of *S. sibirica* are rich in oils.

### 2.3. Thin Layer Chromatography (TLC) Identification

**Figure 9:**
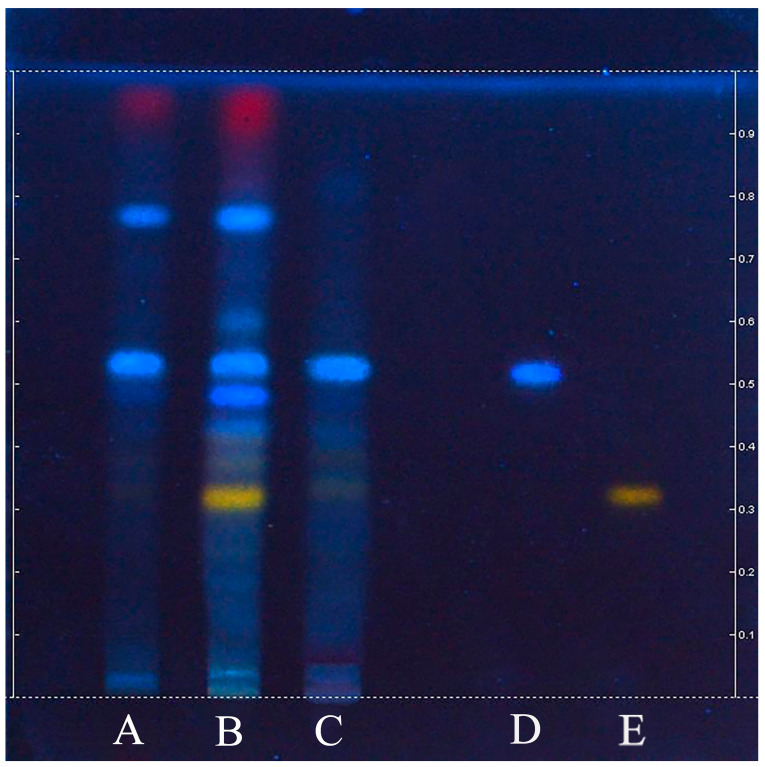
TLC analysis of branches (**A**), leaves (**B**), and fruits (**C**) of *S. sibirica*, and two references: chlorogenic acid (**D**) in bright blue color and rutin (**E**) in light orange color.

TLC is a fundamental analytical technique known for its convenience and speed. It is widely utilized for the identification and analysis of samples according to pharmacopeial standards and drug regulations globally. In Figure 9, a silica gel G thin-layer plate was employed as the stationary phase, whereas the final composition of the developing agent consisted of ethyl acetate, formic acid, and water at a ratio of 9:1.5:1.5 (water-saturated). The separation effect was satisfactory, with no tailing and Rf values ranging from 0.20 to 0.80. The TLC chromatogram shows the results for the branches, leaves, and fruits of *S. sibirica*, with rutin and chlorogenic acid used as reference compounds. Both the reference samples and the three tested samples produced bands of the same color at identical Rf values. The leaves presented the most bands, with Rf values of 0.32 (bright orange, rutin), 0.38 (light orange), 0.42 (light blue), 0.48 (bright blue), 0.52 (bright blue, chlorogenic acid), 0.58 (light blue), and 0.76 (bright blue). The branches displayed bands with Rf values of 0.48 (light blue), 0.32 (bright orange, rutin), and 0.76 (light blue). The fruits presented bands with Rf values of 0.32 (light orange, rutin), 0.42 (light blue), 0.48 (light blue), and 0.52 (light blue, chlorogenic acid). All parts contained chlorogenic acid, although rutin was present exclusively in the leaves. A band with an Rf value of 0.76 was observed in both the leaves and branches but not in the fruits. Although rutin was absent in the branches in this analysis, it was confirmed to be present in the leaves and fruits. These findings indicate that the TLC method established in this study can qualitatively identify rutin and chlorogenic acid in *S. sibirica*, thereby serving as potential quality markers.

### 2.4. Determination of Total Flavonoids

Rutin was chosen as a standard solution. The linear equation for rutin in the concentration range of 0.01 to 0.06 mg·mL^−1^ is y = 11.317x − 0.0449 (r = 0.9997), indicating a good linear relationship between the rutin concentration and absorbance within this range. The absorbance values for the branches, leaves, and fruits of *S. sibirica* were substituted into the calibration curve, yielding total flavonoid contents of 7419.80, 5193.10, and 3629.10 μg·g^−1^ (dry weight) for the leaves, branches, and fruits, respectively. The good precision, reproducibility, stability, and recovery indicated that the method was reliable and suitable for the determination of the total flavonoid content (see Supplementary Materials).

### 2.5. Qualitative and Quantitative Analysis

#### 2.5.1. Qualitative Analysis of Five Analytes in *S. sibirica*

**Figure 10:**
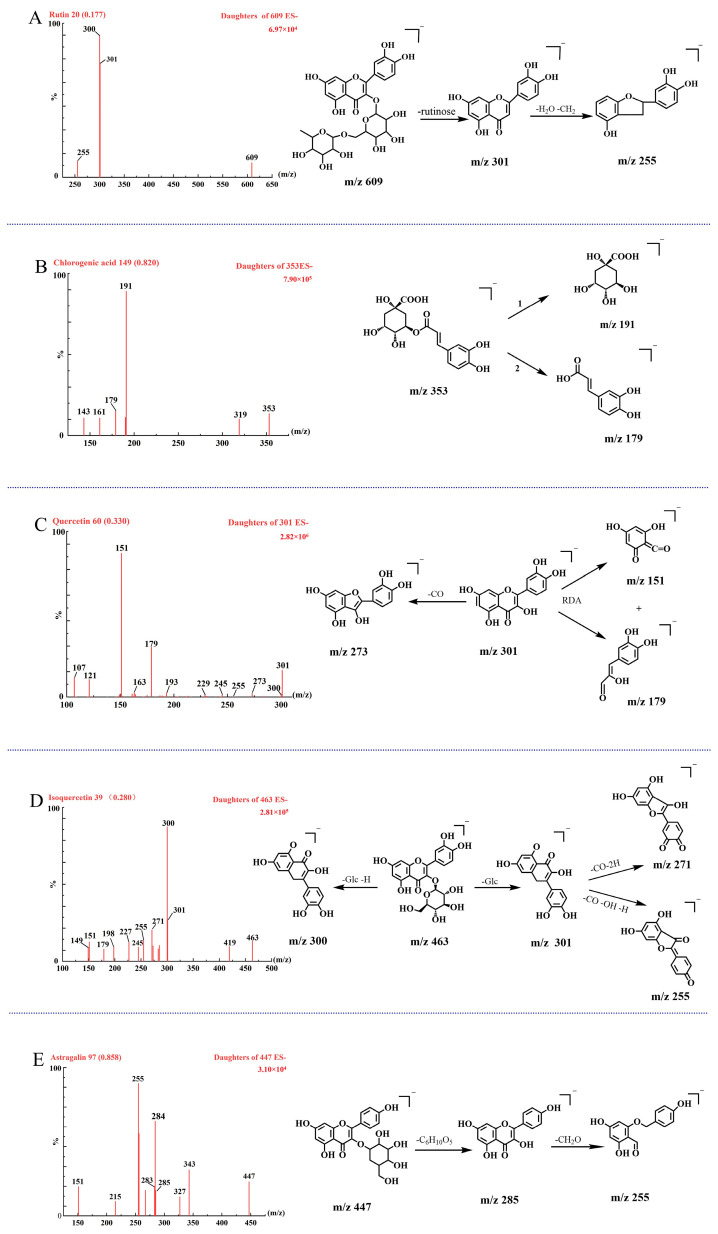
MS/MS spectra of the protonated molecular ions of analytes under the negative ESI mode and their proposed fragmentation patterns. (**A**–**E**) show rutin, chlorogenic acid, quercetin, isoquercetin, and astragalin.

In this study, we analyzed three flavonoid glycosides, one flavone, and one phenolic acid via UPLC-QqQ-MS/MS. We optimized the cone voltage (CV) and collision energy (CE) for each analyte. The mass fragmentation patterns of five analytes were derived based on the obtained MS/MS spectrum and compared with literature [19,20]. For the flavonoid glycosides, the primary mass spectrometry fragmentation pathways observed included the neutral loss of glycosides to produce aglycone ions, the loss of carbon monoxide (CO) from aglycone ions, and retro-Diels–Alder (RDA) fragmentation of the C-ring (Figure 10). The product ion at *m*/*z* 301 was further fragmented to yield a product ion at *m*/*z* 255. For quercetin, the flavone, various proposed MS/MS fragmentation pathways were observed, with the precursor ion detected at *m*/*z* 301 [M-H]^−^ (Figure 10C). The natural loss of CO led to the formation of the product ion at *m*/*z* 273, and further cleavage resulted in product ions at *m*/*z* 151 and *m*/*z* 179 via RDA. The precursor ion of chlorogenic acid was similarly noted at *m*/*z* 353 [M-H]^−^ in negative mode (Figure 10B). The cleavage of the ester bond resulted in a neutral loss of the caffeoyl group, producing a quinic acid ion at *m*/*z* 191. Concurrently, a neutral loss of deoxyquinic acid could occur, yielding product ions of *m*/*z* 179 corresponding to caffeic acid. The most intense ion for each analyte was utilized for multiple-reaction monitoring (MRM) transitions in the quantitative analysis. We optimized the dwell time, cone voltages, and collision energy for each transition to establish a specific and stable MRM method. A summary of all the parameters for each analyte is presented in Table 1. 

#### 2.5.2. A Quantitative Analysis of the Five Analytes in *S. sibirica*

**Figure 11:**
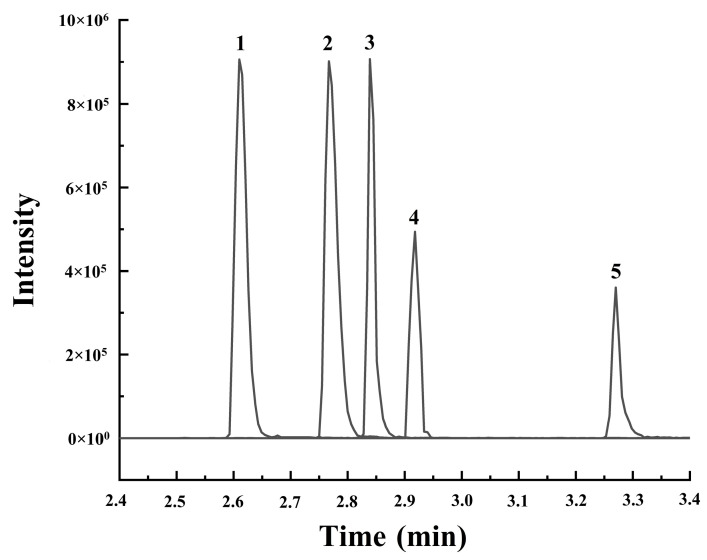
LC-MS/MS MRM chromatograms of 5 analytes’ standard mixtures. 1: chlorogenic acid, 2: rutin, 3: isoquercetin, 4: astragalin, 5: quercetin.

In this study, the UPLC-QqQ-MS/MS method was employed to conduct a quantitative analysis of the five analytes in *S. sibirica*. Typical LC-MS/MS MRM chromatograms of five analytes’ standard mixtures are shown in Figure 11. We validated the parameters of the quantitative method, including linearity, the limit of detection (LOD), limit of quantification (LOQ), precision (both intra-day and inter-day), repeatability, and recovery. The results of the method validation are detailed in Table 2 and Table 3. All calibration curves for the analytes demonstrated excellent linear regression (R^2^ > 0.999) across the tested ranges. The LODs and LOQs for all analytes ranged from 1.0 to 8.5 ng·mL^−1^ and from 3.4 to 28.4 ng·mL^−1^, respectively. The intra-day precision was assessed using six replicates within a single day, whereas the inter-day precision was determined from data collected over three days; both RSD values were less than 1.90%. Stability was evaluated at several time points: 0, 2, 4, 6, 8, 12, 24, and 48 h. The RSDs of the peak areas for all analytes remained below 2.78%, indicating that the test solution was relatively stable throughout the 48 h period. The method’s repeatability was established through six continuous injections of the same sample, yielding an RSD of less than 1.90%. Accuracy was assessed via recovery tests, which involved adding mixed standards at three different concentrations (low, medium, and high concentrations, in triplicate) to the *S. sibirica* samples. The recoveries of the five analytes ranged from 97.53% to 104.70%, with RSD values under 4.41%.

The concentrations of the analytes in the *S. sibirica* samples were calculated from the area of each peak in relation to the regression equations of the standards. As shown in Table 4, the leaves presented the highest content of rutin (4535.60 μg·g^−1^). The final concentrations determined in the branches were 1076.30, 3204.00, 1.60, 13.60, and 22.50 μg·g^−1^, whereas the concentrations in the leaves were 4535.60, 2226.80, 7.50, 74.40, and 20.40 μg·g^−1^, respectively. The concentrations in the fruits were 26.40, 205.70, 1.00, 18.00, and 6.90 μg·g^−1^. This indicates that the order of concentration for the five components is leaves > branches > fruits. 

In previous reports, the contents of various compounds in different *Sambucus* species exhibited significant differences, primarily due to factors such as variety, distribution, environment, climate, grow stage, harvest year, cultivation, and wild type [21,22,23,24,25,26,27,28,29]. Table 5 summarizes the contents of the main components in different *Sambucus* species from various regions. Wang reported that the contents of quercetin and rutin in *S. sibirica* were 0.097–0.198 mg/g and 7.79–13.30 mg·g^−1^ [26]. Seymenska reported high contents of chlorogenic acid ranging from 20.68 to 46.73 mg·g^−1^ in the flowers and leaves of *S. nigra* and *S. ebulus*. Their contents of quercetin were in the range of 0.11–0.92 mg·g^−1^ [22]. They also reported that some *Sambucus* species contained low components. The concentrations of chlorogenic acid, rutin, and quercetin in the fruits of *S. nigra* in Poland and *S. canadensis* ‘Aurea’ and *S. chinensis* in China were less than 530 μg·g^−1^ [21,27]. By comparing these data, it is seen that the contents of components from *S. sibirica* are within the range of *Sambucus* species.

### 2.6. Antioxidant Results 

#### 2.6.1. The Effect of Extracts on the DPPH Scavenging Rate

**Figure 12:**
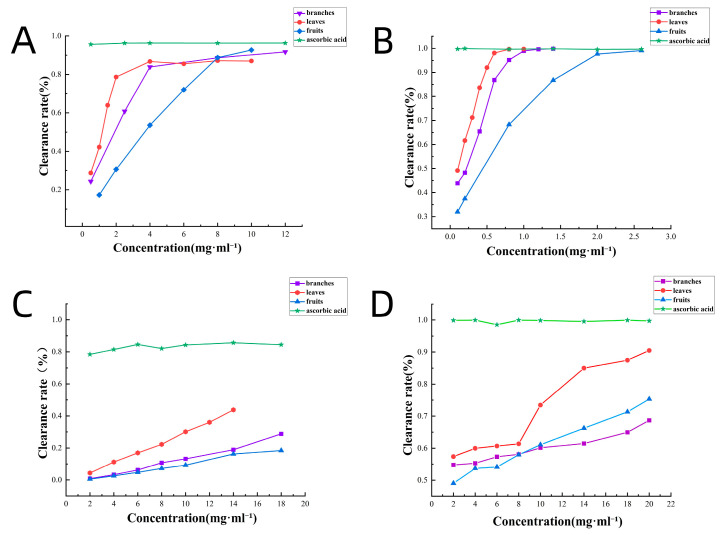
The antioxidant capacity of the extract from *S. sibirica* branches, leaves, and fruits. (**A**) DPPH free radical scavenging activity, (**B**) ABTS free radical scavenging ability, (**C**) ferric reducing antioxidant power, (**D**) superoxide anion free radicals.

The antioxidant capacity of extracts from different parts of *S. sibirica* was determined using the 1,1-diphenyl-2-picryl-hydrazyl (DPPH) method, and the results are shown in Figure 12A; the DPPH radical scavenging activity of the branch, leaf, and fruit extracts showed a concentration-dependent and positively correlated trend within the concentration range of 0.5–12 mg·mL^−1^. The IC_50_ values of the branches, leaves, and fruits were 2.38, 1.33, and 5.18 mg·mL^−1^, respectively. The smaller the IC_50_ value, the stronger the antioxidant activity. Therefore, the scavenging activity of DPPH free radicals was in the order of leaves > branches > fruits.

The antioxidant capacity of extracts from various parts of *S. sibirica* was evaluated using the 1,1-diphenyl-2-picrylhydrazyl (DPPH) method, and the results are presented in Figure 12A. The DPPH radical scavenging activity of the extracts from branches, leaves, and fruits exhibited a concentration-dependent and positively correlated trend within the concentration range of 0.5–12 mg·mL^−1^. The IC_50_ values for the branches, leaves, and fruits were determined to be 2.38, 1.33, and 5.18 mg·mL^−1^, respectively. A smaller IC_50_ value indicates stronger antioxidant activity; thus, the scavenging activity of DPPH free radicals follows the order of leaves > branches > fruits.

#### 2.6.2. The Effects of Extracts on the Clearance of ABTS

As shown in Figure 12B, the 2,2′-azinobis-(3-ethylbenzthiazoline-6-sulphonate) (ABTS) free radical scavenging ability also demonstrated concentration dependence, with leaves exhibiting the strongest clearing ability. The IC_50_ values for the branches, leaves, and fruits were 0.46, 0.32, and 0.84 mg·mL^−1^, respectively. Therefore, the ABTS free radical scavenging ability follows the same order: leaves > branches > fruits.

#### 2.6.3. The Effects of Extracts on the FRAP

As shown in Figure 12C, the antioxidant activity was assessed based on the Fe^3+^ reduction capacity. The results indicate the following order of effectiveness: leaves > branches > fruits.

#### 2.6.4. The Effects of Extracts on the Scavenging Capacity of Superoxide Anion Free Radicals

As illustrated in Figure 12D, the extracts from the branches, leaves, and fruits of *S. sibirica* demonstrated concentration-dependent scavenging activity against superoxide anion radicals. The free radical scavenging activity of the extracts continuously increased within the concentration range of 2–20 mg·mL^−1^.

The antioxidant activity of the three parts is leaves > branches > fruits, which is consistent with the ranking of total flavonoid contents and the total amounts of the five analytes, indicating that the higher the active ingredient, the higher the antioxidant activity. Flavonoids are known for their potent antioxidant properties, which make them capable of scavenging free radicals and inhibiting oxidative stress, thereby protecting cells from damage [2]. And some other components in *S. sibirica* also exhibit antioxidant activity. The fruits of *S. sibirica* contain the lowest amount of flavonoids and the lowest antioxidant activity. However, some *Sambucus* fruits, such as *S. nigra*, provide strong antioxidant activity due to its high anthocyanins [30].

## 3. Materials and Methods

### 3.1. Plant Material

The plant materials used in this study were collected from Altai, Xinjiang, China (47°56′46″ N, 88°07′57″ E), in August 2023. It was authenticated as *Sambucus sibirica* by Prof. Xu Haiyan of Xinjiang Medical University. The voucher specimens were deposited in herbarium of Xinjiang Medical University. Then, the samples were divided into branches, leaves, and fruits.

### 3.2. Chemicals and Reagents

Standard substances for rutin, chlorogenic acid, and quercetin were purchased from the China Institute for the Identification of Pharmaceutical and Biological Products, (Beijing, China), with purities of ≥98%, isoquercetin (≥98%) was purchased from the China Institute for Food and Drug Control, (Beijing, China), and astragalin (≥98%) was purchased from Beijing Solaybao Technology Co., Ltd. (Beijing, China). Petroleum ether, ethyl acetate, anhydrous ethanol, cyclohexane, hydrated chloral, glacial acetic acid, xylene, formaldehyde, and glycerol were purchased from Tianjin Zhiyuan Chemical Reagent Co., Ltd. (Tianjin, China). Potassium bromide was purchased from Tianjin Damao Chemical Reagent Factory, (Tianjin, China), and formic acid was purchased from Fisher Co., USA. (Shanghai, China). Fast Green (CAS: 2353-45-9) and Safranin (CAS: 477-73-6) were from Solarbio Biotechnology Co., Ltd. (Beijing, China). LC-MS-grade acetonitrile was purchased from Merck, Darmstadt, Germany; ascorbic acid was purchased from Chengdu Kelong Chemical Reagent Factory, (Chengdu, China); DPPH (W27F10E81251) was purchased from Shanghai Yuanye Biotechnology Co., Ltd. (Shanghai, China); and enzyme-linked immunosorbent assay was purchased from Thermo Fisher Scientific Technology Co., Ltd. (Shanghai, China). ABTS, potassium ferrocyanide, trichloroacetic acid, and potassium chloride were purchased from Tianjin Shengao Chemical Reagent Co., Ltd. (Tianjin, China).; high-efficiency silica gel G thin-layer plates were purchased from Anhui Liangchen Co., Ltd. (Maanshan, China).; and surgipath paraplast paraffin was purchased from Leica, Germany, (Beijing, China). Sodium hydroxide (analytical grade) was purchased from Tianjin Beilian Fine Chemicals Development Co., Ltd. (Tianjin, China). Aluminum nitrate (≥99%) was purchased from Tianjin Guangfu Fine Chemical Research Institute, (Tianjin, China). Sodium nitrite (analytical grade) was purchased from Tianjin Yongsheng Fine Chemical Co., Ltd. (Tianjin, China). UV-Vis Spectrophotometer, UV-2700, was from Shimadzu Corporation, (Tokyo, Japan).

### 3.3. Preparation of Sample and Standard Solutions

#### 3.3.1. Preparation of Sample Solutions

The branches, leaves, and fruits of *S. sibirica* were crushed via a multifunctional pulverizer from Shanghai Yuanwo Industry & Trade Co., Ltd. (Shanghai, China) and subsequently sieved through a 60-mesh sieve. A precise weight of 1 g of powder from the branches, leaves, and fruits was taken. To this mixture, 30 mL of 80% methanol was added, followed by sonication (100 W, 100 Hz) for 30 min to counteract any weight loss. The mixture was then centrifuged at 13,000× *g* for 10 min at 25 °C to collect the supernatant. Prior to injection, the solution was filtered through a 0.22 μm microporous membrane.

#### 3.3.2. Preparation of Standard Solutions

A mixed standard stock solution containing rutin, chlorogenic acid, quercetin, isoquercetin, and astragalin was prepared in methanol. Working standard solutions were then prepared by diluting the mixed standard solution with methanol to achieve a series of appropriate concentrations: rutin (20–200 μg·mL^−1^), chlorogenic acid (10.0–250.0 μg·mL^−1^), quercetin (0.1–20 μg·mL^−1^), isoquercetin (0.3–5.0 μg·mL^−1^), and astragalin (0.1–1.2 μg·mL^−1^). All standard stock and working solutions were stored at 4 °C before use and filtered through a 0.22 mm microporous membrane prior to injection.

### 3.4. Microscopic Identification of Transverse Sections

The branches of *S. sibirica* were cut into 2–3 cm segments, the leaves were diced into 0.5 × 0.5 cm tissue blocks, and the fruits were halved. Intact and healthy samples were selected and placed in glass bottles with an appropriate amount of formalin-aceto-alcohol (FAA) fixative (38% formaldehyde/glacial acetic acid/70% ethanol, volume ratio of 5:5:90). (1) Fixation: The samples were fixed at room temperature for 3 days. (2) Washing: After fixation, the samples were removed and washed under running water for 36 h. (3) Dehydration: The samples were dehydrated through a series of ethanol solutions with increasing concentrations (30%, 50%, 70%, 80%, 90%, 95%, and 100%), with each solution used for 2 h, followed by 1 h of dehydration in 100% ethanol. (4) Clearing: The samples were then immersed in a series of xylene–ethanol mixtures (1/3 xylene + 2/3 ethanol, 1/2 xylene + 1/2 ethanol, 2/3 xylene + 1/3 ethanol) for 1 h each, followed by 10 min in pure xylene. (5) Infiltration: The samples were infiltrated in a mixture of 1/2 xylene + 1/2 paraffin for 3 h and then in pure paraffin for 4 h. (6) Embedding: After infiltration, the samples were embedded in molten paraffin and allowed to cool and solidify. (7) Sectioning: The paraffin blocks were sectioned into 12 µm thick slices by using a rotary microtome. The sections were then affixed to glass slides and dried in an oven. (8) De-waxing and rehydration: The sections were first immersed in pure xylene, followed by a series of ethanol solutions (1/2 xylene + 1/2 ethanol, pure ethanol, and 95%, 80%, and 70% ethanol), with each solution applied for 5 min for de-waxing and rehydration. (9) Staining: The sections were stained in Bismarck Brown solution for 3.5 h, followed by dehydration through a gradient of ethanol solutions (30%, 50%, 70%, 80%, and 95% ethanol), with each step lasting 2 min. (10) Fast Green Staining: The sections were then stained in Fast Green solution for 10 s, followed by immersion in pure ethanol for 30 s, and finally cleared in xylene. (11) Mounting: Finally, the sections were mounted using resin diluted with xylene [31]. The tissue characteristics of the paraffin sections of S. sibirica branches, leaves, and fruits were observed under a Nikon Eclipse Ni-U upright fluorescence microscope (Nikon Corporation, Tokyo, Japan).

### 3.5. Microscopic Identification of Powders

Temporary slides containing branches, leaves, and fruit powder from *S. sibirica* were prepared. An appropriate amount of powder was placed on a glass slide, followed by the addition of 3–4 drops of chloral hydrate solution, which was mixed evenly. The mixture was gently heated multiple times until the color of the powder became lighter and more thoroughly mixed. After cooling, 2–3 drops of dilute glycerol were added to seal the slide, which was subsequently observed under a microscope [32].

### 3.6. Fourier Transform Infrared Spectroscopy Analysis

The KBr was dried in a 120 °C oven for 48 h and subsequently placed in a drying cylinder before each experiment. The sample powders were dried at 50 °C for 4 h. A total of 2 mg of the powder was combined with 200 mg of KBr, mixed, and finely ground. This mixture was then pressed into transparent tablets, with three replicates prepared for each sample. An FTIR spectrometer (Bruker, Germany) was used to analyze the tablets, and the spectra were recorded over the range of 0–4000 cm^−1^ [33].

### 3.7. Thin-Layer Chromatography Identification

Silica gel G thin-layer plates were used in this study and were activated at 105 °C for 30 min. The plant extracts (2 μL/needle) and the standard solution (1 μL/needle) were subsequently applied onto the thin-layer plate via a semiautomatic sampling device (CAMAG, Muttenz, Switzerland). Nitrogen was utilized as the carrier gas, with a bandwidth of 8 mm and a spot distance of 10 mm from the bottom edge of the thin layer plate. After the samples were applied, the plate was placed in a development cylinder for 40 min to achieve saturation. The developing agent used was a mixture of ethyl acetate, formic acid, and water (9:1.5:1.5 *v*/*v*), which was saturated with water. The development distance of the plate was measured from 10 mm at the bottom edge to 10 mm at the top edge. Following development, a 3% AlCl₃ solution (in ethanol) was sprayed onto the plate, which was then heated for coloration and documented at 366 nm [34].

### 3.8. Determination of Total Flavonoid Content

The total flavonoid content was determined according to the photocolorimetric method [35]. Briefly, 0.5 g of sample consisting of branches, leaves, and fruit powder was weighed and separately extracted with 15 mL of 60% ethanol. All the samples were extracted via ultrasonication (100 W, 100 Hz, and 30 °C for 40 min). The extracts were then centrifuged at 5000× *g* for 10 min, and the supernatant was collected for use in the antioxidant experiment. Each sample was tested in triplicate. The ethanol extract was filtered, and 1 mL of the solution was mixed with 0.3 mL of 5% NaNO_2_, followed by thorough mixing and resting for 6 min. Then, 0.3 mL of 10% Al(NO_3_)_3_ was added, mixed, and allowed to stand for another 6 min. Finally, 4 mL of NaOH solution was added. The final solution volume was adjusted to 10 mL with deionized water (DW). The mixture was allowed to stand at room temperature for 15 min, and the absorbance at 500 nm was measured using a spectrophotometer. A standard calibration curve was plotted using the standard absorbance values and concentrations (10–60 µg/mL) of rutin. The experiment was performed in triplicate.

### 3.9. Quantitative Analysis

#### 3.9.1. UPLC-QqQ-MS/MS Analysis Conditions

Chromatographic conditions: The analytical experiments were carried out on a Waters ACQUITY^®^ UPLC H Class system (Waters, Milford, MA, USA). Chromatographic separation was performed with a Waters ACQUITY UPLC BEH C18 column (50 mm × 2.1 mm, 1.7 μm, Waters, Wexford, Ireland) at 35 °C. The mobile phases were 0.1% formic acid water (A) and acetonitrile (B) using gradient elution. The gradient program was used according to the following profile: 97–97% A in 0–1 min, 97–30% A in 3–4 min, and 30–97% A in 4–6 min. The flow rate was set at 0.3 mL/min, and the injection volume of the test solution was 2 μL.

Mass spectrometry conditions: MS analyses were carried out on a Waters XEVO TQD system (Waters Corp., Milford, MA, USA) equipped with electrospray ionization (ESI). The flow rate of drying gas (N_2_) was 650 L/h, the ion source temperature was 150 °C, the desolventizing gas was nitrogen, the temperature was 350 °C, the flow rate was 50 L/h, the capillary voltage was −2.5 kv; and the acquisition mode was multi-reaction monitoring (MRM) mode.

#### 3.9.2. Method Validation

The calibration curves were generated by plotting the peak areas (y) against the corresponding concentrations of each analyte (x). The LOD and LOQ were determined based on the signal-to-noise ratio, employing the equations LOD = 3 N/S and LOQ = 10 N/S, where N represents the standard deviation of the response and S denotes the slope of the corresponding calibration curve. Intra-day and inter-day precision were assessed by analyzing known concentrations of standard solutions in six replicates within one day, as well as conducting experiments involving triplicates on consecutive days. Six different solutions prepared from the same sample were analyzed to confirm the repeatability of the developed assay. The stability test involved analyzing the sample solution at various time intervals (0, 2, 4, 6, 8, 12, 24, and 48 h), with relative standard deviations (RSDs) of the peak areas of each analyte serving as the measure of stability. Recovery was assessed by adding known amounts of standards at three different concentrations (high, medium, and low) to a sample. The average recovery percentage was calculated using the following formula: recovery (%) = (final concentration − original concentration)/spiked concentration × 100% [16].

### 3.10. Antioxidant Experiment

#### 3.10.1. Preparation of Sample Solution

The preparation process of the sample solution is described in Section 3.6. Each sample was tested in triplicate.

#### 3.10.2. DPPH Free Radical Scavenging Activity

The radical scavenging ability of DPPH was assessed following a method adapted from a previous report [36]. A stock solution of 2 mmol·L^−1^ DPPH was prepared using anhydrous ethanol and subsequently diluted to 0.2 mmol·L^−1^ to create a DPPH working solution. To a 96-well plate, 200 μL of the 0.2 mmol·L^−1^ DPPH solution was added, followed by the addition of 100 μL of *S. sibirica* extracts: branch extracts (0.5, 2.5, 4, 8, 12, 14, 16, 18 mg·mL^−1^), leaf extracts (0.5, 1, 1.5, 2, 4, 6, 8, 10 mg·mL^−1^), and fruit extracts (1, 2, 4, 6, 8, 10, 14, 16 mg·mL^−1^) at varying mass concentrations. After thorough mixing, the plate was incubated in the dark for 30 min. The absorbance value (Ai) of each sample solution was measured at a wavelength of 517 nm using an enzyme-linked immunosorbent assay (ELISA) reader. Anhydrous ethanol was used as a blank for the DPPH solution Aj and for the extract solution Ao, with 3 wells designated for each concentration. L-ascorbic acid served as a positive control in this experiment. The DPPH clearance rate and half-maximal inhibitory concentration (IC_50_) value of the extract were calculated using the following formula: DPPH radical scavenging rate = [1 − (Ai − Aj)/Ao] × 100%.

#### 3.10.3. ABTS Free Radical Scavenging Ability

The free radical scavenging ability of ABTS was evaluated following the methods of Li et al. with slight modifications [37]. Initially, a 7 mmol·L^−1^ ABTS solution was mixed with an equal volume of a 2.5 mmol·L^−1^ potassium persulfate solution and allowed to react in the dark for 16 h to produce the ABTS stock solution. Prior to use, this stock solution was diluted with the appropriate extraction solvent to achieve an absorbance of 0.70–0.80 at 734 nm. Subsequently, 100 μL of extracts from branches, leaves, and fruits at various concentrations was added to a 96-well plate. Next, 200 μL of the ABTS working solution was added and mixed, and the mixture was allowed to stand in the dark at room temperature for 6 min. Ai for each sample was measured at a wavelength of 734 nm. The background absorbance value (Aj) was determined using the corresponding extraction solution in place of the ABTS working solution, whereas distilled water was used to determine the maximum absorbance (Ao). This procedure was repeated three times. Using L-ascorbic acid as a positive control, the ABTS clearance rates and IC_50_ values of the extracts were calculated using the following formula: ABTS free radical scavenging rate = [1 − (Ai − Aj)/Ao] × 100%.

#### 3.10.4. Ferric Reducing Antioxidant Power

To assess the ferric reducing antioxidant power, 100 μL of extracts from branches, leaves, and fruits of *S. sibirica* at different concentrations was prepared following the methods outlined by Jing et al. with slight modifications [38]. For each sample, 250 μL of PBS buffer (pH 6.6) and 250 μL of 0.1% potassium ferrocyanide solution were added sequentially and mixed thoroughly. The mixture was then placed in a water bath at 50 °C for 20 min. Following this incubation process, 250 μL of 10% trichloroacetic acid solution was added and mixed well. The samples were then centrifuged at 4000× *g* for 10 min, and 50 μL of the supernatant was transferred to a 96-well plate. To each well, 50 μL of 0.1% ferric chloride was added, mixed thoroughly, and allowed to stand for an additional 10 min. Ai was measured at 700 nm, using distilled water in place of the sample extract for Ao. Three wells were prepared for each concentration. Using L-ascorbic acid as a positive control, the reducing ability was calculated as A = Ai − Ao, with a higher absorbance indicating a stronger reducing capacity.

#### 3.10.5. Superoxide Anion Free Radicals

The ability of *S. sibirica* extract to scavenge superoxide anion free radicals was assessed following the methods established by Wang et al. with minor modifications [39]. A total of 80 μL of extracts from the branches, leaves, and fruits of *S. sibirica* at various mass concentrations (2, 4, 6, 8, 10, 14, 18, and 20 mg·mL^−1^) was added to each well of a 96-well plate, accompanied by 180 μL of Tris HCl buffer. After thorough mixing, the plate was placed in a water bath set to 25 °C for 20 min. At this point, 20 μL of preheated pyrogallol, maintained at the same temperature, was added. The mixture was well combined, and timing commenced. The reaction continued in a 25 °C water bath for 5 min, after which 40 μL of 10 mmol·L^−1^ HCl was added to terminate the reaction. The absorbance was measured at 325 nm and recorded as A, the absorbance with distilled water replacing pyrogallol was labeled Ai, and the absorbance of the blank (distilled water) was labeled as Aj. L-ascorbic acid was utilized as a positive control, and three replicates were established for each concentration. The clearance rate was calculated using the following formula: R = 1 − (A − Ai)/Aj × 100%.

## 4. Conclusions

In this study, we investigated the micromorphology, phytochemical characteristics, and antioxidant capacity of the branches, leaves, and fruits of *S. sibirica*. We observed cross sections and powders under a microscope at various magnifications, systematically detailing the cross-sectional characteristics of each of these plant parts. Notably, we discovered crystal clusters ranging from 10 to 60 μm in diameter distributed within the sponge tissue of the leaves. The analysis of *S. sibirica* powders revealed various structural features, including different types of ducts, cork cells, non-glandular hairs, oil droplets, stone cells, scale hairs, and star-shaped hairs. FTIR spectra revealed the functional groups present in the samples. Notably, the FTIR spectra of branches and leaves closely resembled each other, indicating a relatively consistent chemical composition, whereas the fruit displayed a distinct absorption peak at 1730 cm^−1^, suggesting a notable lipid content. TLC successfully identified the presence of chlorogenic acid in all three parts, with rutin being particularly evident in the leaves. The total flavonoid contents were determined by a photocolorimetric approach, and a UPLC-QqQ-MS/MS method was established for the qualitative and quantitative analysis of five compounds. The results show the contents of total flavonoids, and five compounds were the highest in the leaves, followed by the branches, with the fruit exhibiting the lowest levels. The branches, leaves, and fruits of *S. sibirica* presented antioxidant activities following the order of effectiveness of leaves > branches > fruits, which aligns with the results of the total flavonoid contents and the quantitative analysis. This study provides valuable insights into the microscopic characteristics, chemical composition, and antioxidant properties of *S. sibirica*; establishes a foundation for future pharmacognosy research and quality standards; and provides a reference for its potential development and utilization.

## Figures and Tables

**Table 1:** Mass spectrometry parameters of the five analytes of *S. sibirica* (in negative ESI mode).

Compound	Formula	Parent Ion (*m*/*z*)	Daughter Ion (*m*/*z*)	Dwell (s)	CV (v)	CE (v)
Rutin	C_27_H_30_O_16_	609	255	0.02	60	50
			300	0.02	60	35
Chlorogenic acid	C_16_H_18_O_9_	353	179	0.02	36	30
			191	0.02	36	20
Quercetin	C_15_H_10_O_7_	301	151	0.02	40	26
			179	0.02	40	22
Isoquercetin	C_21_H_20_O_12_	463	271	0.02	45	45
			300	0.02	45	25
Astragalin	C_21_H_20_O_11_	447	255	0.02	50	44
			284	0.02	50	56

**Table 2:** Linearity, LOD, LOQ, and stability of analytes.

NO.	Analytes	RT (min)	Range (μg·g^−1^)	Regression Equations	R^2^	LOD (ng·mL^−1^)	LOQ (ng·mL^−1^)	48 h Stability (RSD%, *n* = 6)
1	Rutin	2.77	20–200	y = 92.698x + 5512.3	0.9991	4.5	14.9	2.39
2	Chlorogenic acid	2.61	10–250	y = 234.46x + 1679.6	0.9993	3.8	12.5	1.59
3	Quercetin	3.27	0.1–2	y = 1671.2x − 79.086	0.9993	8.5	28.4	1.86
4	Isoquercetin	2.84	0.3–5	y = 394.92x + 68.319	0.9992	3.1	10.2	1.18
5	Astragalin	2.91	0.1–1.2	y = 506.88x − 6.5775	0.9995	1.0	3.4	2.78

**Table 3:** Precision, repeatability, and spiking recovery of analytes.

NO.	Analytes	Precision (RSD%)		Repeatability (RSD%, *n* = 6)	Recovery (RSD%, *n* = 3)					
		Intra-Day (*n* = 6)	Inter-Day (*n* = 6)		High		Middle		Low	
					Mean	RSD%	Mean	RSD%	Mean	RSD%
1	Rutin	1.39	4.39	1.70	97.53	3.04	102.28	2.51	100.92	1.31
2	Chlorogenic acid	1.59	2.89	1.09	102.20	2.46	100.98	1.82	104.70	2.41
3	Quercetin	2.78	4.70	1.90	99.94	0.23	103.56	4.41	99.79	1.52
4	Isoquercetin	1.19	3.74	1.76	99.76	1.20	97.88	0.82	101.86	1.08
5	Astragalin	1.59	2.89	1.78	101.80	2.80	99.71	1.16	104.91	0.81

**Table 4:** Contents of five analytes in branches, leaves, and fruits of *S. sibirica*.

Analytes	Contents (Mean ± SD, *n* = 3, μg·g^−1^ in Dry Weight)		
	Leaves	Branches	Fruits
Rutin	4535.60 ± 0.02	1076.30 ± 0.05	26.40 ± 0.05
Chlorogenic acid	2226.80 ± 0.01	3204.00 ± 0.02	205.70 ± 0.02
Quercetin	7.50 ± 0.02	1.60 ± 0.05	1.00 ± 0.05
Isoquercetin	74.40 ± 0.02	13.60 ± 0.03	18.00 ± 0.03
Astragalin	20.40 ± 0.02	22.50 ± 0.04	6.90 ± 0.04

**Table 5:** The contents of flavonoids and phenolic acid compounds in different *Sambucus* from various regions.

Species	Regions (Type)	Parts	Methods	Contents				Units ^a^	Ref.
				Chlorogenic Acid	Rutin	Quercetin	Isoquercetin		
*S. nigra*	Poland (unknown)	fruits	HPLC-UV	48.8–7.2	18.9–333.6	20.0–221.7	—	μg·g^−1^ DW	[21]
*S. nigra*	Bulgaria (wild)	flowers	HPLC-UV/Vis	46.733	—	0.116	—	mg·g^−1^ DW	[22]
		leaves		21.491	—	0.178	—		
*S. ebulus*		flowers		23.075	—	0.164	—		
		leaves		20.680	—	0.921	—		
*S. nigra* subsp. *canadensis*	East of the USA (wild)	fruits	HPLC-UV	0.107	0.525	0.022	0.065	mg·g^−1^ FW	[23]
*S. williamsii*	Shandong, China (cultivate)	root bark	HPLC-UV	—	0.026–0.033	0.007–3.570	—	mg·g^−1^ DW	[24]
		branches			0.100–0.340	0.090–0.360			
		leaves			1.860–4.090	1.240–3.570			
		fruits			0.130–0.430	0.060–0.720			
*S. williamsii*	Jilin, China (wild)	branches	HPLC	— — —	0.171 0.522 0.165	— — —	— — —	mg·g^−1^ DW	[25]
		leaves							
		fruits							
*S. sibirica*	Xinjiang, China (wild)	branches and leaves	HPLC-UV	—	7.785–13.300	0.097–0.199	—	mg·g^−1^ DW	[26]
*S. canadensis ‘Aurea’*	Ningxia, China (cultivate)	branches	HPLC-UV	—	660	0.480	—	μg·g^−1^ DW	[27]
		leaves		—	530	nd	—		
*S. chinensis*	China (unknown)	branches and leaves	HPLC-UV	53.9–346.0	—	—	—	μg·g^−1^ DW	[28]
*S. formosana*	Taiwan, China (unknown)	whole plant	HPLC-DAD-MS	2.7	4.8	6.4	3.9	mg·g^−1^ DW	[29]

^a^ DW: dry weight; FW: fresh weight.

## Data Availability

The raw data supporting the conclusions of this article will be made available by the authors upon request.

## Supplementary Materials

The following supporting information can be downloaded at https://www.mdpi.com/article/10.3390/molecules29235503/s1, Validation of determination of total flavonoid content. Figure S1. Rutin standard curve. Table S1. Precision, repeatability, and stability of total flavonoid measurement by UV-Vis spectrophotometry. Table S2. Spike recovery data.
