# Supplementary material for: Microscopic Identification, Phytochemical Analysis, and Study of Antioxidant Properties of Branches, Leaves, and Fruits of Kazakh Medicine Sambucus sibirica
Source: Molecules. 2024 Nov 21;29(23):5503. doi: 10.3390/molecules29235503 (PMC11643177; doi:10.3390/molecules29235503)
Supplement: Supplementary file 1 [file molecules-29-05503-s001.zip › molecules-3284371-supplementary.pdf]

Supplementary Materials

1. Validation of determination of total flavonoid content

The linear equation for rutin in the concentration range of 0.01 to 0.06 mg·mL^{-1} is y = 11.317x-0.0449 (r=0.9997), indicating a good linear relationship between the rutin concentration and absorbance within this range（**Figure S1**）. The absorbance values for the branches, leaves, and fruits of *S. sibirica* were substituted into the calibration curve, yielding total flavonoid contents of 7.42 mg·g^{-1} DW for the leaves, 5.19 mg·g^{-1} DW for the branches, and 3.63 mg·g^{-1} DW for the fruits. Precision measurements were taken by pipetting 6 aliquots of 2.5 mL standard solution, and the absorbance was measured by UV-Vis spectrophotometry according to the method described in section "3.9.2", resulting in an average absorbance of 0.524 with RSD=1.46% (n=6), demonstrating good instrument precision. Similarly, 6 aliquots of 2 mL sample solution were pipetted into 10 mL volumetric flasks, and 60% ethanol was added to the mark. The samples were reacted according to the procedure in section "3.9.2" and their absorbance values ranged from 0.395 to 0.412, with an average of 0.405 and RSD=1.14% (n=6), indicating good reproducibility of the method. The stability of the rutin standard solution's color reaction was also tested by measuring the absorbance at 5 min intervals within 30 min, with absorbance values ranging from 0.403 to 0.423, an average of 0.414, and RSD = 1.23% (n = 6), confirming that the color reaction of rutin is stable within 30 min (**Table S1**). For the recovery tests, 6 aliquots of 1.0 mL leaf sample extracts (with a known concentration of 0.049 mg·mL^{-1}) were transferred into 10 mL volumetric flasks, divided into three groups, and spiked with 0.15, 0.25, and 0.4 mL of standard solution, respectively. After mixing and color development according to the procedure in section "3.9.2", the absorbance at 500 nm was measured. The average recovery rate was 99.32%, with a relative standard deviation (RSD) of 2.49%, indicating that the method is reliable and suitable for the determination of total flavonoid content (**Table S2**).

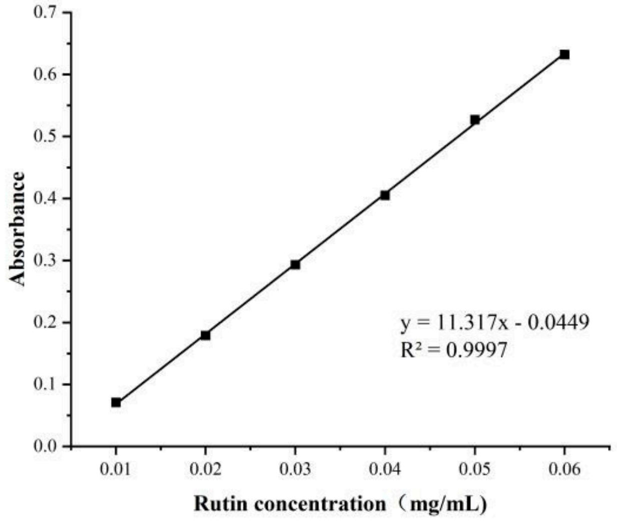

Figure S1. Rutin Standard Curve

Table S1. Precision, Repeatability, and Stability of Total Flavonoid Measurement by UV-Vis Spectrophotometry

	Precision		Repeatability		Stability	
	Content (mg)	Absorbance	Content (mg)	Absorbance	Content (mg)	Absorbance
1	0.05	0.528	0.04	0.402	0.04	0.423
2	0.05	0.525	0.04	0.412	0.04	0.422
3	0.05	0.521	0.04	0.411	0.04	0.414
4	0.05	0.513	0.04	0.401	0.04	0.412
5	0.05	0.522	0.04	0.395	0.04	0.409
6	0.05	0.536	0.04	0.412	0.04	0.403
Mean	0.05	0.524	0.04	0.405	0.04	0.414
RSD		1.46%		1.14%		1.23%

Table S2. Spike Recovery Data

NO.	Original content (mg)	Added amount (mg)	Theoretical value (mg)	Measured value (mg)	Recovery rate (%)	Mean recovery rate (%)	RSD (%)
1	0.049	0.03	0.079	0.078	97.96	99.32	2.49
2	0.049	0.03	0.079	0.079	100		
3	0.049	0.05	0.099	0.098	97.96		
4	0.049	0.05	0.099	0.101	104.81		
5	0.049	0.08	0.129	0.128	98.96		
6	0.049	0.08	0.129	0.128	97.96		
